# Novel MicroRNA-Regulated Transcript Networks Are Associated with Chemotherapy Response in Ovarian Cancer

**DOI:** 10.3390/ijms23094875

**Published:** 2022-04-28

**Authors:** Danai G. Topouza, Jihoon Choi, Sean Nesdoly, Anastasiya Tarnouskaya, Christopher J. B. Nicol, Qing Ling Duan

**Affiliations:** 1 Department of Biomedical and Molecular Sciences, Queen’s University, 18 Stuart St., Kingston, ON K7L 3N6, Canada; danai.topouza@queensu.ca (D.G.T.); jihoon.choi@queensu.ca (J.C.); nicolc@queensu.ca (C.J.B.N.); 2 School of Computing, Queen’s University, 21-25 Union St., Kingston, ON K7L 2N8, Canada; srnesdoly@gmail.com (S.N.); tarnousk@gmail.com (A.T.); 3 Department of Pathology and Molecular Medicine, Queen’s University, 88 Stuart St., Kingston, ON K7L 3N6, Canada; 4 Division of Cancer Biology and Genetics, Queen’s University Cancer Research Institute, Queen’s University, 10 Stuart St., Kingston, ON K7L 3N6, Canada

**Keywords:** platinum-based chemotherapy resistance, high-grade serous ovarian cancer, The Cancer Genome Atlas, differential expression analysis, coexpression network analysis, microRNAs, expression quantitative trait loci

## Abstract

High-grade serous ovarian cancer (HGSOC) is a highly lethal gynecologic cancer, in part due to resistance to platinum-based chemotherapy reported among 20% of patients. This study aims to generate novel hypotheses of the biological mechanisms underlying chemotherapy resistance, which remain poorly understood. Differential expression analyses of mRNA- and microRNA-sequencing data from HGSOC patients of The Cancer Genome Atlas identified 21 microRNAs associated with angiogenesis and 196 mRNAs enriched for adaptive immunity and translation. Coexpression network analysis identified three microRNA networks associated with chemotherapy response enriched for lipoprotein transport and oncogenic pathways, as well as two mRNA networks enriched for ubiquitination and lipid metabolism. These network modules were replicated in two independent ovarian cancer cohorts. Moreover, integrative analyses of the mRNA/microRNA sequencing and single-nucleotide polymorphisms (SNPs) revealed potential regulation of significant mRNA transcripts by microRNAs and SNPs (expression quantitative trait loci). Thus, we report novel transcriptional networks and biological pathways associated with resistance to platinum-based chemotherapy in HGSOC patients. These results expand our understanding of the effector networks and regulators of chemotherapy response, which will help to improve the management of ovarian cancer.

## 1. Introduction

High-grade serous ovarian cancer (HGSOC) is a highly lethal gynecologic cancer, in part due to resistance to first-line, platinum-based chemotherapy treatment among 20% of patients [1]. Chemotherapy-resistant patients have a significantly shorter overall survival (OS) than sensitive patients, and many experience tumor recurrence within six months of completing chemotherapy [2]. There is currently no strategy for predicting response to platinum-based chemotherapy, which reflects our limited understanding of the underlying molecular mechanisms of chemotherapy resistance [3].

MicroRNAs (miRNAs) are small single-stranded noncoding RNAs that post-transcriptionally repress mRNA expression and are involved in the regulation of all biological processes and diseases [4], including ovarian cancer pathogenesis and chemotherapy response [5]. MiRNA and gene expression profiling of patient tumors has the potential to identify signatures that determine disease prognosis [6]. Such prognostic signatures have been implemented in clinic for some cancers [7], but no such tests exist for ovarian cancer patients to date. The detection of transcriptomic signatures of progression-free survival and chemotherapy resistance is an area of active interest, and several previous studies have defined mRNA and miRNA signatures of chemotherapy resistance in HGSOC patients.

The majority of earlier studies reporting genes associated with platinum-based chemotherapy resistance in ovarian cancer patients had employed univariate analysis methods on transcriptomics data [8]. These methods assume that chemotherapy response is driven by a single gene. It is well established, however, that chemotherapy response, like other drug-response outcomes, is a complex multifactorial trait modulated by multiple genes contributing to common biological pathways [9,10]. To date, few studies have investigated chemotherapy response in ovarian cancer using multivariate, machine-learning methods to generate novel hypotheses for the underlying biological mechanisms [11,12,13,14,15]. Fewer still have incorporated multiple types of omics datasets such as mRNA and miRNA sequencing in addition to genomics data to further investigate the regulation of associated gene networks [16,17]. Finally, earlier studies that employed multivariate methods had used expression microarray data, which do not allow for discovery of novel transcript isoforms [18,19]. In contrast, RNA-sequencing data using next-generation technology include gene transcripts that may have been missed by traditional microarray profiling.

In this study, we apply both univariate and multivariate analysis methods to high-throughput miRNA- and mRNA-sequencing data from tumors of HGSOC patients to identify novel biological pathways and networks associated with chemotherapy response. We further determine miRNAs and single-nucleotide polymorphisms (i.e., expression quantitative trait loci, eQTLs) correlated with the expression of the associated transcriptional networks. These findings are validated in two independent ovarian cancer cohorts and improve our understanding of the biological mechanisms underlying resistance to platinum-based chemotherapy in HGSOC patients.

## 2. Results

### 2.1. Study Design

**Figure 1:**
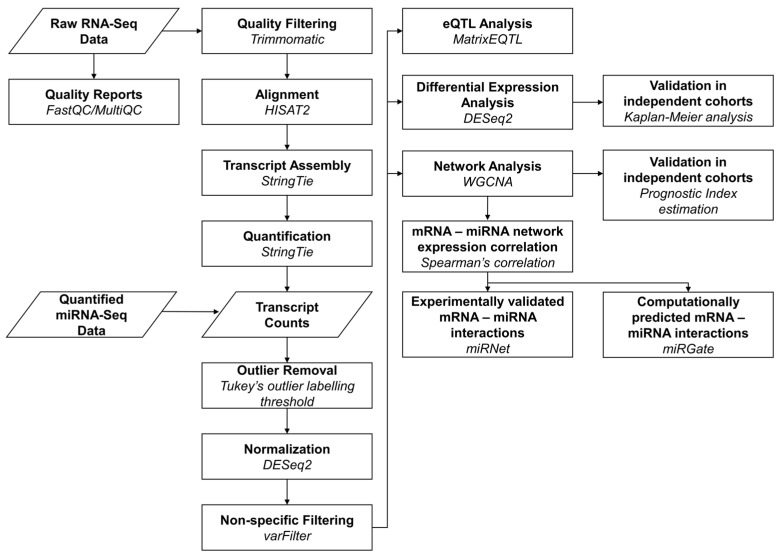
Flowchart of study analysis pipeline. First, reports of read-sequence quality were generated using FastQC [20] and MultiQC [21], followed by trimming of low-quality mRNA-seq reads with Trimmomatic [22]. Filtered reads were aligned to the hg19 human reference genome [23] using HISAT2 (hierarchical indexing for spliced alignment of transcripts) [24] and quantified by StringTie [25]. miRNA-seq data were obtained as quantified reads from the Cancer Genome Atlas [26]. Next, transcript expression was normalized using the DESeq2 R package [27], and highly variable transcripts were selected with the varFilter function of the genefilter R package [28]. Moreover, transcript expression was used to test for differential expression using DESeq2 and to construct coexpression networks using the weighted correlation network analysis (WGCNA) R package [29]. Interactions between miRNAs and mRNAs from significant WGCNA networks were detected using Sperman’s correlation, and validated using miRNet [30] and miRGate [31]. Validation of differential expression analysis results was performed using Kaplan–Meier analysis on two independent HGSOC cohorts. Validation of network analysis results was performed using the Prognostic Index estimation method from the SurvExpress tool [32] on the same validation cohorts. Transcript expression data were integrated with genomics data from the same patients to determine eQTLs (expression quantitative trait loci) using the MatrixEQTL R package [33].

We obtained miRNA- and mRNA-sequencing data from HGSOC patients of The Cancer Genome Atlas (TCGA). An analysis pipeline for these data was curated as summarized in Figure 1 and detailed in the Methods and Appendix A. Our pipeline applied select software and tools for quality control of raw sequence reads, alignment to a reference genome, quantification of reads into transcript isoform expression values, outlier removal, and normalization. We separated the TCGA patients into chemotherapy-sensitive and chemotherapy-resistant groups based on their platinum-free interval (Methods). After preprocessing, we performed differential expression and network coexpression analyses, as well as integration of miRNAs, mRNAs, and eQTLs. Our results were validated in two independent HGSOC cohorts.

### 2.2. Differentially Expressed miRNAs and mRNAs between Sensitive and Resistant Patients

**Figure 2:**
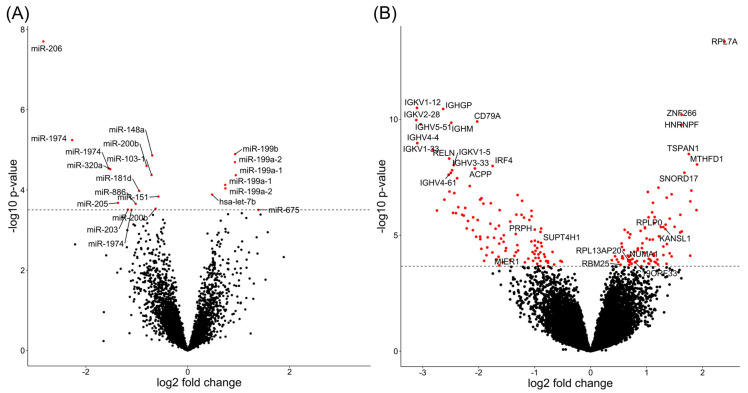
Differential expression analysis of miRNA and mRNA transcripts. (**A**) Significant differentially expressed miRNA isoforms (*n* = 21) are shown in red. The Benjamini–Hochberg adjusted significance threshold is indicated by the dashed line. Transcripts with a positive fold change are upregulated in resistant patients, whereas transcripts with a negative fold change are downregulated in resistant patients. (**B**) Significant differentially expressed mRNA transcripts (*n* = 196) are shown in red. The Benjamini–Hochberg adjusted significance threshold is indicated by the dashed line. Transcripts with a positive fold change are upregulated in resistant patients, whereas transcripts with a negative fold change are downregulated in resistant patients. Gene symbol labels are provided for the top 20 most significant mRNAs, as well as for mRNAs that were replicated in an independent cohort (Results Section 2.7, Table S11).

Differential miRNA expression analysis revealed 21 differentially expressed miRNA isoforms between chemotherapy-sensitive and -resistant patients (adjusted *p* < 0.05), which map to 16 unique miRNAs (Figure 2A, Table S1). Pathway enrichment analysis of these miRNA isoforms revealed 16 pathways, such as blood vessel morphogenesis and negative regulation of autophagy (Table S2).

Differential expression analysis identified 196 mRNAs associated with chemotherapy response (adjusted *p* < 0.05) that map to 190 unique genes (Figure 2B, Table S3). Pathway enrichment analysis of these associated transcripts indicated enrichment of 41 annotation terms, including B-cell receptor regulation, complement activation, and peptide chain elongation (Table S2).

### 2.3. miRNA Coexpression Networks Involved in Lipid Transport and Oncogenic Pathways Associated with Chemotherapy Response

**Figure 3:**
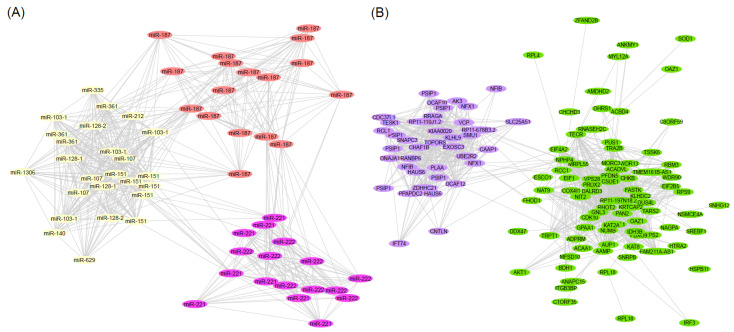
Weighted correlation network analysis of mRNA and miRNA transcripts. (**A**) The miRNA modules *lightcoral*, *plum*, and *ivory* are visualized in their respective colors. Each node represents one miRNA isoform, and each edge represents a connection or coexpression. The distance between nodes is the connection weight, where more similarly expressed transcripts are plotted closer. (**B**) The mRNA modules *lavenderblush3* and *darkolivegreen* are visualized in their respective colors. Each node represents a transcript, and each edge represents a connection or coexpression. The distance between nodes is the connection weight, where more similar transcripts are plotted closer.

Weighted gene coexpression network analysis (WGCNA) of the miRNA dataset constructed 100 coexpression modules (Table S4), of which three are associated with chemotherapy response. The *ivory* (*p* = 0.0098, log OR = −5.67) and *lightcoral* (*p* = 0.042, log OR = −4.36) modules are negatively associated with chemotherapy resistance, while the third *plum* network (*p* = 0.045, log OR = 4.29) is positively associated with chemotherapy resistance. The *ivory* network consisted of 25 miRNA isoforms mapping to 11 unique miRNAs (Figure 3A, Table S5), which are enriched for seven pathways and functions, including regulation of lipoprotein transport and cholesterol efflux (Table S2). The *lightcoral* module consists of 17 isoforms of miR-187 and the *plum* network consists of 17 isoforms of miR-221 and miR-222 (Figure 3A, Table S5). While no pathway annotations were derived for the *lighcoral* module, the *plum* module is enriched for 18 pathways and oncogenic functions, such as inhibition of the TRAIL-activated apoptotic pathway and inflammatory cytokine production, and upregulation of protein kinase B signaling (Table S2).

### 2.4. mRNA Coexpression Networks Involved in Protein Ubiquitination and Fatty-Acid Metabolism Associated with Chemotherapy Response

WGCNA of the mRNA transcripts resulted in 58 coexpression modules, of which 2 were associated with platinum-based chemotherapy (Table S6). First, the *lavenderblush3* module is negatively associated with chemotherapy resistance (*p* = 0.016, log OR = −5.40). This module contains 39 transcripts, mapping to 31 unique genes (Figure 3B, Table S7). Pathway analysis indicates enrichment of biological pathways related to protein ubiquitination, and the binding motif for the transcription factor GABP-alpha (Table S2). Second, the *darkolivegreen* module is significantly upregulated in chemoresistant patients (*p* = 0.032, log OR = 13.63) and contains 82 transcripts mapping to 80 unique genes (Figure 3B, Table S7). This module is significantly enriched for the protein-containing complex term, as well as for eight pathways involved in fatty-acid metabolism with nominal significance (Table S2).

### 2.5. Germline eQTLs May Regulate the Expression of Associated miRNAs, mRNAs, and Networks

Integrative analysis with germline SNP data identified 268 unique *cis*-eQTLs associated with the expression of significant mRNAs and miRNAs (Table S8). A total of 20 SNPs are associated with the expression of 7 significant miRNAs, and 248 SNPs are associated with the expression of 55 significant mRNAs. Of the 268 SNPs, 118 are novel eQTLs, whereas 126 are previously known and 24 are not yet recorded in the annotation database. The majority (227) are predicted to alter regulatory motifs, and 67 are associated with 94 human phenotypes from published genome-wide association studies. The most common phenotypes are related to triglycerides, high-density lipoprotein (HDL), and low-density lipoprotein (LDL) cholesterol.

### 2.6. Network Integration Reveals miRNA-Mediated Regulation of Chemotherapy Response Mechanisms

**Figure 4:**
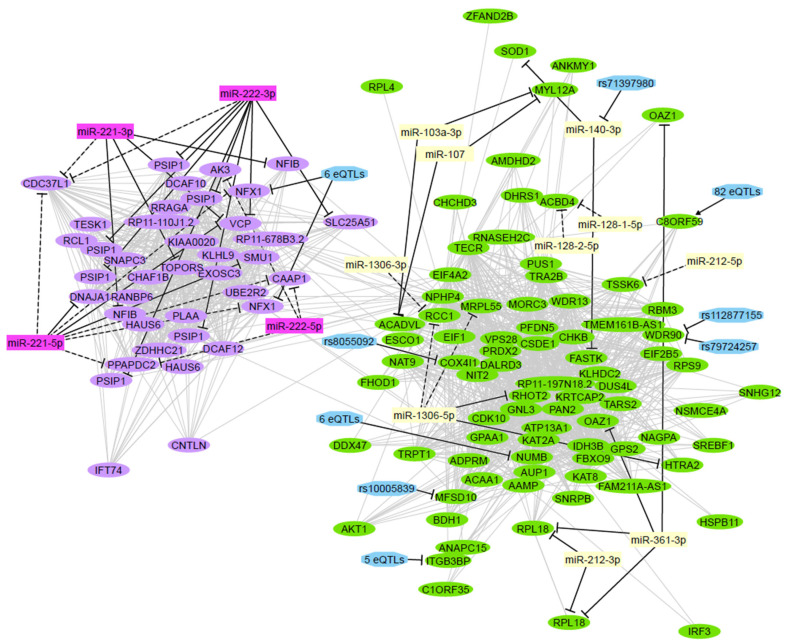
Integration of significant networks. The mRNA transcripts (oval nodes) from the *lavenderblush3* and *darkolivegreen* modules are arranged based on coexpression similarity (grey edges). Regulatory interactions (black edges) indicate inhibition (bar-headed arrow) or activation (arrow). The colors of miRNA nodes indicate their membership in the *plum* and *ivory* coexpression networks. Four miRNA isoforms (rectangular nodes) were identified, either using experimental methods (solid edges) or in silico predictions (dashed edges), to regulate the expression of genes in the *lavenderblush3* network module. In addition, 6 eQTLs (hexagonal nodes) may regulate the expression of the NFX1 gene. Genes in the *darkolivegreen* module have 10 validated or predicted regulatory miRNAs and 97 regulatory eQTLs. Finally, the *ivory* miRNA miR-140 may be regulated by the miR-QTL rs71397980. eQTL, expression quantitative trait loci; miR-QTL, microRNA expression quantitative trait loci.

Integration of the associated miRNA and mRNA networks determined that the *plum* miRNA network significantly correlates with the *lavenderblush3* mRNA network (Spearman correlation of module eigengenes, ρ = −0.26, *p* < 0.001), and the *ivory* miRNA network significantly correlates with the *darkolivegreen* mRNA network (Spearman correlation of module eigengenes, ρ = −0.17, *p* = 0.023). Annotations using miRNet and miRGate determined that 20 of these mRNA-miRNA interactions are experimentally validated, while 15 others are supported by in silico predictions (Table 1). The experiments that validated miRNA binding to mRNA molecules involved HITS-CLIP (high-throughput sequencing of RNA isolated by crosslinking immunoprecipitation), AGO-CLIP (Argonaute-crosslinking and immunoprecipitation), CLASH (crosslinking, ligation, and sequencing of hybrids), and PAR-CLIP (photoactivatable ribonucleoside-enhanced crosslinking and immunoprecipitation) assays (Table S9). The computational prediction algorithms inferred miRNA-mRNA binding by assessing the complementarity between the miRNA seed sequence and the mRNA transcript, as well as the mRNA-miRNA duplex energy (Table S9). Combined with the potential cis-eQTL regulation of mRNAs and miRNAs in these networks, these results reveal an integrative, multi-omics view of transcriptional networks associated with chemotherapy response in ovarian cancer (Figure 4).

### 2.7. Replication in Two Independent Ovarian Cancer Cohorts

**Figure 5:**
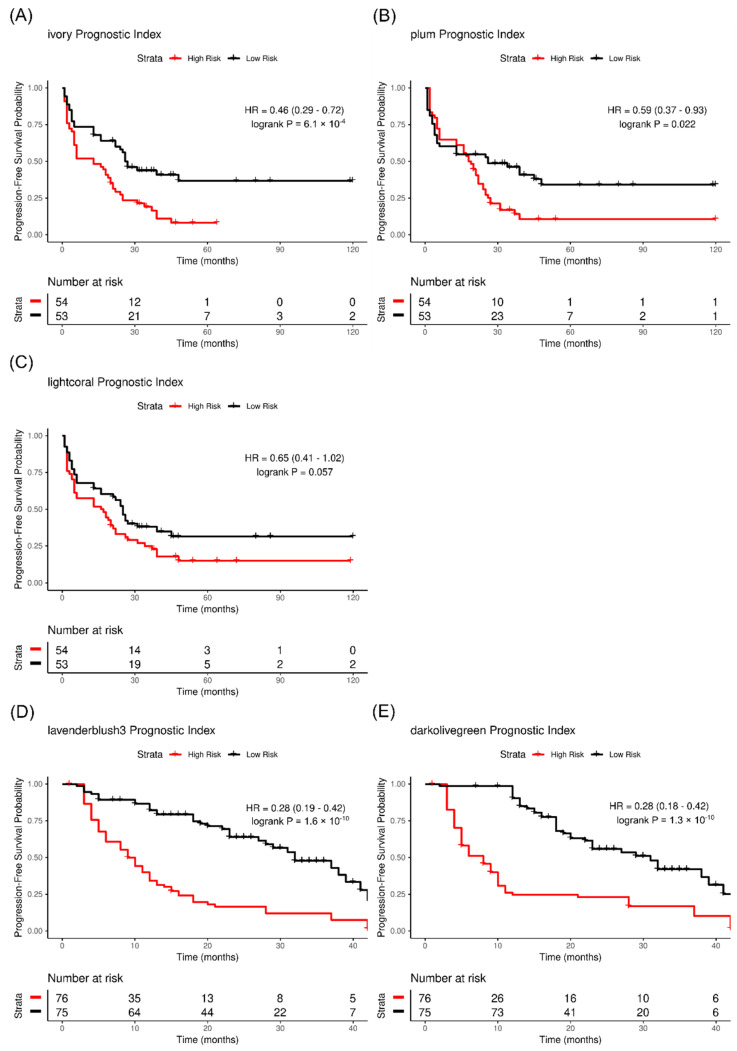
Replication analysis of significant transcript coexpression networks. (**A**–**C**) Kaplan–Meier analysis of patient PFS association with the prognostic index of miRNA networks *ivory*, *plum*, and *lightcoral* in the MITO cohort. The *ivory* (**A**) and *plum* (**B**) miRNA network modules replicated in the MITO cohort (*p* = 6.1 × 10^−4^, log HR = −0.78 and *p* = 0.022, log HR = −0.52, respectively), while the *lightcoral* module (**C**) reached nominal significance (*p* = 0.057, log HR = −0.43). (**D**,**E**) Kaplan–Meier analysis of patient PFS association with the prognostic index of mRNA networks *lavenderblush3* and *darkolivegreen* in the AOCS cohort. The *lavenderblush3* (**D**) and *darkolivegreen* (**E**) mRNA network modules replicated in the AOCS cohort (*p* = 1.6 × 10^−10^, log HR = −1.27 and *p* = 1.3 × 10^−10^, log HR = −1.27, respectively). PFS, progression-free survival; AOCS, Australian Ovarian Cancer Study; MITO, Multicenter Italian Trial in Ovarian cancer.

Replication of the differentially expressed miRNAs used miRNA-seq data from the MITO cohort (Table S10). The *ivory* and *plum* miRNA network modules replicated in the MITO cohort (*p* = 6.1 × 10^−4^, log HR = −0.78 and *p* = 0.022, log HR = −0.52, respectively), while the *lightcoral* module reached nominal significance (*p* = 0.057, log HR = −0.43) (Figure 5A–C).

Replication analysis of the differentially expressed mRNAs used RNA microarray data from the AOCS cohort (Table S11). The *lavenderblush3* and *darkolivegreen* mRNA network modules replicated in the AOCS cohort (*p* = 1.6 × 10^−10^, log HR = −1.27 and *p* = 1.3 × 10^−10^, log HR = −1.27, respectively) (Figure 5D,E).

## 3. Discussion

In this study, we identified miRNA and mRNA transcripts and networks associated with chemotherapy response in HGSOC patients. Our findings implicate novel and known biological pathways that were further replicated in independent cancer cohorts. In addition, we identified potential interactions among these miRNA and mRNA networks, as well as eQTLs that potentially regulate the associated transcripts. Thus, our results provide an integrative, multi-omics view of biological networks associated with chemotherapy response in HGSOC.

We identified one miRNA coexpression network (*ivory*), associated with chemotherapy sensitivity in HGSOC, which is involved in the negative regulation of lipid transport. This enrichment is mainly mediated by miR-128-1 and miR-128-2, which play a key role in cholesterol and lipid homeostasis through their suppression of the ABCA1 cholesterol efflux transporter and the low-density lipoprotein receptor (LDLR) [43,44]. MiR-148a, which is significantly downregulated in resistant patients, is also a regulator of these key genes [43]. The overexpression of *ABCA1* is associated with reduced survival in OC patients [45], and levels of LDLR are increased in chemoresistant OC cell lines [46]. In addition, overexpression of miR-128 promotes sensitivity to cisplatin in previously resistant OC cells [47]. Our results are consistent with the chemosensitivity-promoting role of miR-128 and its potential activity in cholesterol efflux inhibition alongside miR-148a in this cohort.

We identified a second miRNA coexpression network (*plum*) consisting of miR-221 and miR-222 isoforms, which have been implicated in the development of chemotherapy resistance in OC. Expression of miR-221/miR-222 transcripts is high in cisplatin-resistant OC cell lines, and their inhibition increases cellular sensitivity [48]. Overexpression of miR-221 and miR-222 promotes proliferation of OC cell lines [49,50] and is associated with reduced disease-free and overall survival [49]. Thus, our findings are consistent with earlier studies showing increased activity of miR-221 and miR-222 in chemoresistant tumors.

The *lavenderblush* mRNA coexpression network was significantly upregulated in platinum-sensitive patients, which replicated in another independent cohort (AOCS). This module consists of genes involved in ubiquitin-mediated proteolysis in the endoplasmic reticulum (ER). We also detected a significant downregulation of genes responsible for translation initiation in sensitive patients in our differential mRNA expression analysis. These findings suggest that the unfolded protein response (UPR), a cellular process responsible for resolving ER stress, may be increasingly activated in sensitive patients compared to resistant cases. The UPR alleviates ER stress through several pathways, including increased ER-associated protein degradation (ERAD) to remove misfolded proteins, and inhibition of translation to reduce protein load in the ER [51]. ER stress promotes cisplatin resistance in OC cell lines [52] and the upregulation of ERAD genes such as *VCP* in the *lavenderblush3* module is associated with longer OS and platinum sensitivity in HGSOC cohorts [17,53]. Finally, the *lavenderblush3* genes *VCP*, *DNAJA1*, and *TOPORS* are overexpressed in platinum-sensitive HGSOC patients as part of a cell cycle and damage-response-associated network [16].

The second mRNA coexpression network associated with chemotherapy resistance in our HGSOC cohort that replicated in the AOCS (*darkolivegreen*) included genes associated with fatty-acid metabolism (*SREBF1*, *ACAA1*, *ACADVL*), and the protein kinase B oncogene (*AKT1*), which promotes de novo lipid biosynthesis in cancer [54]. SREBF1 is a key enzyme for cholesterol and fatty-acid synthesis, and an essential gene for OC tumor growth [55]. Specifically, *SREBF1* is activated by *AKT1*, promoting fatty-acid synthesis [56], which favors cell proliferation in OC [57]. Expression of *ACADVL*, involved in the β-oxidation of long-chain fatty acids, is linked to OC metastasis and cell survival [58]. Our findings indicate the upregulation of these lipid metabolism genes among chemotherapy-resistant patients. Lipid metabolism dysregulation activates the UPR, which triggers lipid metabolism-based adaptations in the cell through several pathways, including *SREBF1* regulation [59]. The interaction of these pathways may present a link between our two gene coexpression modules and warrants further study.

Differential expression analysis identified a downregulation of mRNA transcripts involved in the adaptive immune system, which is associated with chemoresistance. Previous studies reported that a high tumor immune score is a strong predictor of chemosensitivity in HGSOC [60]. In addition, there are potential links between this immune response activation, UPR, and lipid metabolism. ER stress can induce proinflammatory cytokine production and UPR activation in tumor cells [61], which can disrupt dendritic cell function in the OC tumor microenvironment [62]. Moreover, dendritic cell function can also be inhibited by increased lipid uptake in various cancers [63].

Integrative analysis of mRNA-seq and miRNA-seq datasets identified potential interactions of the associated transcript coexpression modules. The overexpression of miR-221/222 in resistant patients may be inhibiting the chemosensitivity-associated *lavenderblush3* mRNA network, revealing a novel potential mechanism of chemotherapy resistance. This finding, combined with the accumulating evidence of miR-221/miR-222 involvement in chemoresistance, may point to a promising avenue for therapeutic intervention. However, overexpression of miR-221/miR-222 promotes UPR-induced apoptosis in hepatocellular carcinoma (HCC) cells [64]. Additionally, ER stress suppresses miR-221/miR-222 in HCC, promoting resistance to apoptosis. The contribution of this mechanism to chemotherapy response in HGSOC is currently unclear and presents an area for future investigation.

We also identified potential regulation of the *darkolivegreen* mRNA module by the *ivory* miRNA network, which may inhibit lipid metabolism in chemotherapy-sensitive patients. As increased lipid metabolism by cancer cells is a known mechanism of chemoresistance in HGSOC, this miRNA-mediated inhibition may present a novel mechanism of chemotherapy sensitivity.

Finally, *cis*-eQTL analysis identified known and novel genomic variants correlated with the expression of mRNAs and miRNAs, which are associated with lipid-related phenotypes. These SNPs have not been previously associated with platinum-based chemotherapy response in ovarian cancer [65] and may represent novel genomic associations with chemotherapy response. High HDL and triglyceride levels have been correlated with increased cancer stage at diagnosis in OC patients [66]. In addition, advanced-stage OC patients with high LDL levels have a shorter PFS than patients with normal levels [67]. Further investigation of these eQTLs is necessary to further elucidate their role in platinum-based chemotherapy resistance and HGSOC prognosis.

To date, there have been few studies to investigate miRNA and mRNA network associations with chemotherapy response in ovarian cancer using multivariate analysis methods. Bernardini et al. (2005) used unsupervised two-dimensional hierarchical clustering and feature selection to identify genes that were predictive of response to platinum chemotherapy [12]. Spentzos et al. (2005) used pattern recognition and compound covariate predictive algorithms that identified a multigene pattern to classify patient chemotherapy response [11]. Bagnoli et al. (2016) applied a semisupervised principal component analysis method on selected miRNAs, leading to a prognostic miRNA model whose expression is associated with risk of disease progression [15]. Chen et al. (2018) used WGCNA to identify gene networks associated with chemosensitivity [13]. Zhang et al. (2019) also used WGCNA and identified gene networks associated with chemoresistance [14]. The above studies all performed multivariate analysis using a single data modality; two additional studies made use of multiple omics datasets to investigate chemotherapy response. Benvenuto et al. (2020) used a micrographite algorithm to integrate significant mRNA and miRNA expression profiles into a single network that distinguished chemotherapy-sensitive and -resistant patients [16]. Finally, our group previously used WGCNA to identify gene networks associated with chemotherapy sensitivity; the expression of significant genes was integrated with patient germline genomic polymorphisms to identify cis-eQTLs that may regulate the expression of these networks [17]. All of the above studies used expression data from microarrays, which do not allow for the distinction of miRNAs from the same sequence family and of mRNA transcript isoforms [18,19]. Our use of miRNA- and mRNA-sequencing data, as well as our analysis of three different data modalities, results in a more detailed profiling of the transcriptome and generates novel hypotheses for the biological mechanisms underlying variable chemotherapy response in ovarian cancer.

Our study provides novel insight of the underlying mechanisms modulating resistance to platinum-based chemotherapy in HGSOC. Specifically, we conducted whole-transcriptome analysis of miRNA-seq and mRNA-seq data to generate novel mechanistic hypotheses using both univariate and network methods. Moreover, we integrated this data with miRNA-seq and genome-wide SNPs to determine potential regulation of the associated transcripts and networks. Our findings implicate novel and known signaling pathways and networks associated with chemotherapy response in HGSOC as well as regulators, which could become novel drug targets. Further studies are needed to validate these findings in other cancers, and to investigate the contribution of these networks to patients’ overall survival.

## 4. Materials and Methods

### 4.1. Chemotherapy Response Classification

Sequencing of miRNA and mRNA was derived from chemotherapy-naïve tumors of 191 and 205 HGSOC patients of TCGA, respectively [68]. Patients who received platinum-based adjuvant chemotherapy were selected and classified for chemotherapy response based on their platinum-free interval. Sensitive patients remained cancer-free for at least 12 months after chemotherapy completion, whereas resistant patients experienced cancer recurrence within 6 months (Table 2; Appendix A).

### 4.2. Processing of Sequencing Data

The miRNA-sequencing data were downloaded as quantified expression files (level 3 data from TCGA). Sequencing reads from mRNA were downloaded as FASTQ files (level 1 data from TCGA), filtered for base-quality, aligned, and quantified (detailed in Appendix A). Both mRNA and miRNA datasets underwent outlier detection, normalization, and nonspecific filtering, resulting in 49,116 mRNA and 4479 miRNA transcripts for further analyses.

### 4.3. Differential Expression Analysis

Differentially expressed miRNA and mRNA transcripts were detected between sensitive and resistant patients using a negative binomial generalized linear model (GLM) in the DESeq2 R package [27]. DESeq2 uses the median-of-ratios normalization method, which divides transcript counts in each sample by a size factor determined by the ratios of gene counts in the sample to the average gene counts among all samples [69]. This method considers the sequencing depth and RNA composition in each sample and is a recommended normalization method for RNA-sequencing data [70,71]. This analysis controlled for patients’ ages at diagnosis, as resistant patients were significantly older (Table 2). The Benjamini–Hochberg method corrected for multiple testing.

### 4.4. Weighted Network Correlation Analysis

The weighted gene coexpression network analysis (WGCNA) R package [29] was used to identify modules of coexpressed miRNA and mRNA transcripts using an unsupervised machine-learning approach. We performed multivariate WGCNA to evaluate the association of miRNA and mRNA networks with chemotherapy response. This method groups coexpressed transcripts into modules prior to testing for association to the clinical outcome. This analysis identifies groups of transcripts that individually may have modest effects on chemotherapy response, but collectively contribute to a common biological network or pathway that is significantly associated with the outcome. In addition, this method reduces the sequencing datasets into a smaller number of transcript modules and uses Principal Component Analysis (PCA) to further summarize the information of each cluster into a representative value, referred to as the eigengene, for association testing. This reduces the multiple testing corrections needed. Module eigengenes were used to determine association with chemotherapy response using a GLM, adjusted for patients’ age as a covariate (Appendix A).

### 4.5. Pathway Enrichment Analysis

Pathway enrichment analysis with g:Profiler [72] was used to determine overrepresentation of biological pathways from lists of differentially expressed transcripts (miRNA and mRNAs) and coexpression networks (Appendix A).

### 4.6. Expression Quantitative Trait Locus Analysis

Germline single-nucleotide polymorphisms (SNPs) from TCGA–HGSOC patients were imputed as described by Choi et al. [17] before undergoing quality control and linkage disequilibrium-based pruning, retaining 1,722,608 common SNPs for analysis. SNPs were integrated with patient miRNA-seq data (*n* = 178) and mRNA-seq data (*n* = 167) to identify correlations with transcript expression (eQTLs) using the MatrixEQTL R package [33] (Appendix A).

### 4.7. mRNA-microRNA Interaction Analysis

Potential regulation of mRNA networks by miRNAs was tested on a subset of patients with both mRNA and miRNA data from the same tumor (*n* = 165). We measured the Spearman correlation of module eigengenes from the mRNA and miRNA coexpression networks, as well as the Spearman correlation of individual mRNA and miRNA transcript expression. Results were validated using miRNet [30], a database of experimentally validated mRNA-miRNA interactions, and miRGate [31], a tool that identifies predicted mRNA-miRNA interactions based on the consensus of several algorithms that assess the complementarity between miRNA seed sequences and mRNA transcript sequences, as well as the mRNA-miRNA duplex energies.

### 4.8. Replication Cohorts and Analysis

Our results were replicated using two independent ovarian cancer cohorts. First, miRNA results were replicated in the Multicenter Italian Trial in Ovarian cancer cohort (MITO; GSE25204; *n* = 130) [73]. Next, mRNA results were replicated in the Australian Ovarian Cancer Study cohort (AOCS; GSE9891; *n* = 285) [74]. Replication of differentially expressed transcripts used the auto-cutoff Kaplan–Meier analysis method from the KM Plotter tool [75] to test the association of each transcript with progression-free survival (PFS) in the AOCS and MITO cohorts. Validation of transcript networks used the Prognostic Index estimation method from the SurvExpress tool [32] to test the association of miRNA and mRNA networks with PFS in the above cohorts (Appendix A).

### 4.9. Software and Statistical Analysis

All statistical analyses were performed using R (v. 3.6.0) [76] in the RStudio environment (v. 1.1.383) [77]. The association tests of continuous patient clinical data, such as age, with chemotherapy-response-employed t-tests, while categorical patient clinical data, such as cancer stage and tumor subtype, were tested with Fisher’s exact tests and Chi-squared tests (stats R package, v. 3.6.0) [76]. Differential expression analyses were performed using DESeq2 (v. 1.26.0) [27] and plotted with ggplot2 (v. 3.3.5) [78] and ggrepel (v. 0.9.1) [79]. Coexpressed transcript networks were detected using WGCNA (v. 1.69) [29] and plotted with Cytoscape (v. 3.7.0) [80]. The association of transcript networks with chemotherapy response was tested using a generalized linear model (stats R package, v. 3.6.0). The correlation of miRNA and mRNA transcript expression was tested using Spearman’s correlation (stats R package, v. 3.6.0). Detection of eQTLs was performed using an additive linear model in the MatrixEQTL R package (v. 2.3) [33]. Validation of differentially expressed transcripts in independent cohorts was performed using Kaplan–Meier analysis with the survival R package (v. 3.2-7) [81] and plotted with survplots from the R package rms (v. 6.1-1) [82]. Validation of network analysis results in independent cohorts was performed using Cox proportional hazards regression models to generate a prognostic index estimator for each network (survival R package, v. 3.2-7).

## Figures and Tables

**Table 1:** Predicted and validated mRNA—miRNA network interactions.

Gene ID	mRNA ID	miRNA ID	Spearman’s ρ	*p* Value	Interaction Evidence
*lavenderblush3* mRNA—*plum* miRNA network interactions					
*PSIP1*	ENST00000397519	hsa-mir-222-3p	−0.32	0.000021	Experimental validation [34]
*CDC37L1*	ENST00000381854	hsa-mir-222-3p	−0.31	0.000042	Computational prediction
*CDC37L1*	ENST00000381854	hsa-mir-221-3p	−0.27	0.00034	Computational prediction
*CDC37L1*	ENST00000381854	hsa-mir-221-5p	−0.27	0.00034	Computational prediction
*NFIB*	ENST00000380959	hsa-mir-221-3p	−0.27	0.00039	Experimental validation [35]
*SMU1*	ENST00000397149	hsa-mir-221-5p	−0.26	0.00082	Experimental validation [36]
*VCP*	ENST00000358901	hsa-mir-222-3p	−0.25	0.00089	Experimental validation [37]
*CAAP1*	ENST00000333916	hsa-mir-222-5p	−0.22	0.0041	Computational prediction
*CAAP1*	ENST00000333916	hsa-mir-221-5p	−0.21	0.0076	Computational prediction
*TOPORS*	ENST00000360538	hsa-mir-221-3p	−0.19	0.014	Experimental validation [38]
*SLC25A51*	ENST00000496760	hsa-mir-222-3p	−0.19	0.016	Experimental validation [37]
*PPAPDC2*	ENST00000381883	hsa-mir-222-5p	−0.18	0.021	Computational prediction
*VCP*	ENST00000358901	hsa-mir-221-3p	−0.17	0.023	Experimental validation [39]
*VCP*	ENST00000358901	hsa-mir-221-5p	−0.17	0.023	Experimental validation [38]
*NFX1*	ENST00000379540	hsa-mir-221-5p	−0.17	0.027	Computational prediction
*PPAPDC2*	ENST00000381883	hsa-mir-221-5p	−0.17	0.03	Computational prediction
*DNAJA1*	ENST00000330899	hsa-mir-221-5p	−0.16	0.035	Experimental validation [38]
*AK3*	ENST00000381809	hsa-mir-222-5p	−0.16	0.037	Computational prediction
*darkolivegreen* mRNA—*ivory* miRNA interactions					
*RPL18*	ENST00000552347	hsa-mir-212-3p	−0.29	0.00017	Experimental Validation [40]
*MRPL55*	ENST00000411464	hsa-miR-1306-5p	−0.25	0.001	Computational prediction
*FASTK*	ENST00000466855	hsa-mir-140-3p	−0.25	0.0013	Experimental Validation [41]
*RPL18*	ENST00000552347	hsa-mir-361-3p	−0.24	0.0021	Experimental Validation [40]
*ACBD4*	ENST00000321854	hsa-miR-128-2-5p	−0.22	0.0041	Computational prediction
*OAZ1*	ENST00000581150	hsa-mir-361-3p	−0.22	0.0047	Experimental Validation [36,40]
*ACBD4*	ENST00000321854	hsa-miR-128-1-5p	−0.21	0.0059	Computational prediction
*MYL12A*	ENST00000578611	hsa-mir-103a-3p	−0.17	0.023	Experimental Validation [38,40]
*MYL12A*	ENST00000578611	hsa-mir-107	−0.17	0.023	Experimental Validation [38,40,42]
*HTRA2*	ENST00000484352	hsa-mir-1306-5p	−0.17	0.029	Experimental Validation [40]
*ACADVL*	ENST00000579425	hsa-mir-103a-3p	−0.17	0.03	Experimental Validation [38]
*ACADVL*	ENST00000579425	hsa-mir-107	−0.17	0.03	Experimental Validation [38]
*SOD1*	ENST00000470944	hsa-mir-140-3p	−0.16	0.037	Experimental Validation [34]
*RHOT2*	ENST00000569675	hsa-mir-1306-5p	−0.16	0.044	Experimental Validation [40]
*TSSK6*	ENST00000360913	hsa-miR-212-5p	−0.15	0.048	Computational prediction
*RCC1*	ENST00000373832	hsa-miR-1306-3p	−0.15	0.048	Computational prediction
*RCC1*	ENST00000373832	hsa-miR-1306-5p	−0.15	0.048	Computational prediction

Both (100%) of the miRNAs in the *plum* network significantly interact with 12 of the 31 (39%) genes in the *lavenderblush* mRNA network. A total of 8 of the 11 (73%) miRNAs in the *ivory* network significantly interact with 12 of the 80 (15%) genes in the *darkolivegreen* mRNA network.

**Table 2:** Characteristics of the HGSOC cohorts with mRNA-Seq and miRNA-Seq data from TCGA.

	mRNA-Seq Cohort				miRNA-Seq Cohort			
	Sensitive	Resistant	*p* Value	All Cases	Sensitive	Resistant	*p* Value	All Cases
Age								
Mean	57.2	62.0	0.0043 ^a^	59.2	57.8	61.5	0.018 ^a^	59.3
Range	30–87	38–87		30–87	30–87	38–87		30–87
Grade								
G2	18 (15.9%)	8 (10.3%)	0.27 ^b^	26 (13.6%)	23 (18.7%)	8 (9.8%)	0.14 ^b^	31 (15.1%)
G3/4	95 (84.1%)	69 (88.5%)		164 (85.9%)	99 (80.5%)	73 (89.0%)		172 (83.9%)
Ungraded	0 (0.0%)	1 (1.3%)		1 (0.5%)	1 (0.8%)	1 (1.2%)		2 (1.0%)
Stage								
II	7 (6.2%)	2 (2.6%)	0.48 ^b^	9 (4.7%)	8 (6.5%)	2 (2.4%)	0.41 ^b^	10 (4.9%)
III	91 (80.5%)	67 (85.9%)		158 (82.7%)	99 (80.5%)	67 (81.7%)		166 (81.0%)
IV	15 (13.3%)	9 (11.5%)		24 (12.6%)	16 (13.0%)	13 (15.9%)		29 (14.2%)
Cytoreductive surgery outcome								
Optimal (≤10 mm)	73 (64.6%)	54 (69.2%)	0.23 ^c^	127 (66.5%)	77 (62.6%)	56 (68.3%)	0.21 ^c^	133 (64.9%)
Suboptimal (>10 mm)	26 (23.0%)	20 (25.6%)		46 (24.1%)	29 (23.6%)	21 (25.6%)		50 (24.4%)
Unknown	14 (12.4%)	4 (5.1%)		18 (9.4%)	17 (13.8%)	5 (6.1%)		22 (10.7%)
Overall survival								
Mortality events	48 (42.5%)	60 (76.9%)	2.35 × 10^−6 c^	108 (56.5%)	55 (44.7%)	66 (80.5%)	0.0002 ^c^	121 (59.0%)
Median months	52.0	28.2	6.97 × 10^−10 a^	39.9	51.9	28.2	1.13 × 10^−10 a^	39.5
95% CI	50.1–54.0	26.2–30.2		37.9–41.9	49.9–53.9	26.2–30.2		37.5–41.5
Adjuvant chemotherapy regimen								
Platinum agent only	4 (3.5%)	3 (3.9%)	1 ^b^	7 (3.7%)	4 (3.3%)	4 (4.9%)	0.71 ^b^	8 (3.9%)
Platinum and Taxane combination	109 (96.5%)	75 (96.2%)		184 (96.3%)	119 (96.8%)	78 (95.1%)		197 (96.1%)
Primary tumor mRNA subtype								
Differentiated	37 (32.7%)	22 (28.2%)	0.49 ^b^	59 (30.9%)	42 (34.2%)	24 (29.3%)	0.68 ^c^	66 (32.2%)
Immunoreactive	23 (20.4%)	10 (12.8%)		33 (17.3%)	24 (19.5%)	13 (15.9%)		37 (18.1%)
Mesenchymal	22 (19.5%)	18 (23.1%)		40 (20.9%)	24 (19.5%)	18 (22.0%)		42 (20.5%)
Proliferative	30 (26.6%)	27 (34.6%)		57 (29.8%)	33 (26.8%)	27 (32.9%)		60 (29.3%)
Unknown	1 (0.9%)	1 (1.3%)		2 (1.1%)	0 (0.0%)	0 (0.0%)		0 (0.0%)
Total patients	113	78		191	123	82		205

^a^—*p*-value based on t-test; ^b^—*p*-value based on Fisher’s Exact test; ^c^—*p*-value based on Chi-Squared test; CI, Confidence Interval.

## Data Availability

Transcriptomics, genomics, and clinical data from the TCGA cohort supporting the conclusions of this article are available in the Genomic Data Commons (GDC) Data Portal at https://portal.gdc.cancer.gov/, project ID TCGA-OV. Clinical and gene expression data of the AOCS cohort supporting the conclusions of this article are available in the Gene Expression Omnibus (GEO) database at https://www.ncbi.nlm.nih.gov/geo/, reference number GSE9899. Clinical and miRNA expression data of the MITO cohort supporting the conclusions of this article are available in the GEO database at https://www.ncbi.nlm.nih.gov/geo/, reference number GSE25204.

## Supplementary Materials

The following supporting information can be downloaded at: https://www.mdpi.com/article/10.3390/ijms23094875/s1.

## Appendix A. Supplementary Methods

### Appendix A.1. Chemotherapy Response Classification

Sensitive patients remained cancer-free one year after completion of chemotherapy, whereas resistant patients experienced cancer recurrence within 6 months of chemotherapy completion. We excluded patients who developed new tumors between six months to one year from chemotherapy completion to enrich for genetic differences between the drug response groups. Included patients received platinum-based adjuvant chemotherapy, which consisted of a platinum agent (3.66–3.90% of patients) or a combination of a platinum agent and a taxane (96.10–96.34% of patients) (Table 2). We excluded patients who were treated with an alternative therapy instead of platinum-based adjuvant chemotherapy, patients without a tumor recurrence who passed away within six months of chemotherapy completion, patients who were followed for less than one year after chemotherapy completion, and patients without transcriptomic data.

### Appendix A.2. RNA-Sequencing Data Preprocessing

Raw mRNA-sequencing reads from frozen chemotherapy-naïve tumor samples of high-grade serous ovarian cancer (HGSOC) patients were obtained from the TCGA database [68] on 31 August 2017 using the TCGAbiolinks R package [83]. Patient mRNA-seq data were filtered for base quality using the Trimmomatic software package [22]. Bases were evaluated in a window of four bases progressing from the 5′ end of each read to the 3′ end. Each read was trimmed when the average quality within the window dropped below 15 on the PHRED base 33 quality scale, which indicates a 3.1% probability of error [84]. Sequences matching Illumina HiSeq adapters and Illumina PCR primers were removed.

Patient RNA-sequence reads were aligned to the hg19 reference genome [85] using the HISAT2 software [24], with a mean overall alignment rate of 96.38%. The aligned reads were assembled into transcripts by the StringTie software package [25] and quantified using the GRCh37.87 Ensembl transcript list (release 92) [86], resulting in the quantification of 196,464 gene transcripts and isoforms for each patient.

Aligned and quantified frozen chemotherapy-naïve tumor microRNA-sequencing data (Level 3) for the TCGA HGSOC cohort were obtained from the Broad Institute Genome Data Analysis Center Firehose [26] (http://gdac.broadinstitute.org) on 17 September 2018 (TCGA data version 2016_01_28 for OV). Patient data were acquired in the form of quantified microRNA-seq isoform counts (*n* = 29,382) in the .txt format. The microRNA (miRNA) isoform data had been aligned to GRCh36/hg18 and quantified using TCGA’s modified version of the British Columbia Genome Sciences Centre miRNA profiling pipeline [87].

### Appendix A.3. Patient-Level Quality Control

Patient mRNA data were iteratively filtered for outliers based on Spearman’s correlation of gene expression between patients, using Tukey’s outlier labeling threshold (Q1—α * IQR) [88] and fine-tuning the α value based on our sample size (α = 2.3) [89] as described by Panarelli et al. [90]. This process removed four patients. Patient miRNA expression profiles were also filtered for outliers using the above method, with an α value of 2.4 based on the larger number of patients in the miRNA cohort. This process removed nine patients.

### Appendix A.4. Transcript-Level Quality Control

Transcript count data were normalized using the median of ratios method from the DESeq2 R software package [27]. Next, we applied nonspecific filtering to the mRNA expression dataset using median absolute deviation (MAD) [28], and retained the top 25% of mRNA transcripts with the highest variance among patients for further analysis (*n* = 49,116). As for nonspecific filtering of the miRNA dataset, we omitted isoforms with variance below the median value, resulting in 14,691 remaining miRNA transcripts. In addition, we removed miRNA isoforms with low expression among patients (isoform count mean < 1), resulting in 4479 miRNAs for further analysis.

### Appendix A.5. Differential Expression Analysis

Volcano plot figures were generated using the R packages ggplot2 (v. 3.3.5) [78] and ggrepel (v 0.9.1) [79].

### Appendix A.6. Pathway Enrichment Analysis

Three differentially expressed miRNAs (miR-151a, miR-103a-1, miR-203a) were re-annotated with the newest miRbase [22] identifiers before pathway analysis with g:Profiler [72]. A total of 3 of the 16 miRNAs (miR-320a, miR-1974, and miR-886) are not yet identified in the g:Profiler database and not included in the pathway enrichment analysis.

### Appendix A.7. Weighted Correlation Network Analysis

Patient transcript expression data were analyzed using the weighted correlation network analysis (WGCNA) R package [29]. We used this tool to calculate the Pearson correlation between all transcripts, then used an adjacency function to form a network where each node is a gene, and each connecting edge corresponds to the strength of the correlation between nodes. The power adjacency function raised the correlations to a power β, making the connectivity distribution of our gene expression network approach that of a scale-free network. Fine tuning indicated β = 10 to be optimal for the mRNA dataset, and β = 9 to be optimal for the miRNA dataset. We constructed a Topological Overlap Matrix (TOM), where the topological overlap of two transcripts is a function of their adjacency and number of shared connections. The TOM-based dissimilarity was then used in hierarchical clustering to generate gene modules. We used a minimum module size of 30 to encourage larger gene clusters, as recommended by the WGCNA manual [91]. After clustering, we merged modules that were not sufficiently distinct from each other to produce robust networks that better capture biological pathways. We calculated a module membership value for each transcript that shows how well a transcript fits into each of the available modules. A total of 50% of transcripts from each module were tested for membership. If more than 25% of transcripts tested had a higher membership value for a module other than their own, those two modules were merged. This process was performed in rounds until no more modules could be merged. Finally, principal component analysis was conducted for all transcripts within a module, resulting in a value called an eigengene. We used these module eigengene values and the patient age covariate to construct a generalized linear model that revealed modules with significant correlation with chemotherapy response. Networks with a significant association were functionally annotated using pathway analysis. Cytoscape was used for network visualization [80].

### Appendix A.8. Expression Quantitative Trait Locus (eQTL) Analysis

Germline single-nucleotide-polymorphism (SNP) data were profiled in normal tissues of HGSOC patients in the TCGA cohort using the Affymetrix SNP Array 6.0. A total of 47,960,330 genetic polymorphisms for 262 patients were obtained after phasing and imputing as described by Choi et al. [17]. The imputed data were then processed for quality control using PLINK 1.9 [92]. We first performed patient-level quality control: 20 patients were removed due to low (F < −0.05) or excessive (F > 0.05) heterozygosity, and 2 patients were removed due to high genetic relatedness (pi-hat > 0.9), leaving 240 patients for further analysis. We then performed variant-level quality control, starting with linkage disequilibrium (LD)-based variant pruning. This process evaluated variants in a window of 50 SNPs, which shifted by 5 SNPs after every iteration, and removed variants with a variance inflation factor (VIF) larger than 2 (r^2^ > 0.5). A total of 7,075,175 independent SNPs were retained after LD pruning. After checking for allelic independence with the Hardy–Weinberg equilibrium, 112 more variants were removed. Finally, we retained SNPs with a minor allele frequency ≥ 5% and variant missingness < 10%, resulting in 1,722,608 variants to be used for further analysis. A total of 167 patients from the RNA-Seq cohort and 178 patients from the miRNA-Seq cohort had genomic data available following quality control.

Next, we tested the association of common patient polymorphisms (*n* = 1,722,608) with mRNA transcript expression (*n* = 196,464) or miRNA isoform expression (*n* = 29,382), in order to determine novel expression quantitative trait loci (eQTLs) in our dataset. We used the MatrixEQTL R package [33] to test SNPs located within 1 Mb of recorded transcripts [93] (*cis*-eQTLs) for association with transcript expression. Based on this distance, 1,676,365 common SNPs were tested for association with mRNA expression, while 356,189 common SNPs were tested for association with miRNA expression. miRNA isoform locations were converted to hg19 coordinates using UCSC’s liftOver utility [94].

We identified 121,900 significantly associated SNP-mRNA transcript pairs and 36,995 significantly associated SNP-miRNA isoform pairs after false-discovery-rate correction. Of those, 248 SNPs were associated with 55 mRNA transcripts found to be significant in differential expression or network analysis, while 20 SNPs were associated with 7 miRNAs that were significant in differential expression or network analysis (Table S8). We mapped these SNPs to an rsID using the dbSNP Build150 Human Variation Set [95], and functionally annotated them using HaploReg v4.1 [96].

### Appendix A.9. Replication Analysis

The replication of our findings was performed in two independent ovarian cancer cohorts The replication of mRNA results was performed on tumor transcriptomic data collected on the Affymetrix Human Genome U133 Plus 2.0 expression microarray from a cohort of 285 patients from the Australian Ovarian Cancer Study (AOCS; GSE9891) [74]. The replication of miRNA results was performed on normalized tumor miRNA data collected on the Illumina Human v2 MicroRNA expression beadchip microarray from a cohort of 130 patients from the Multicenter Italian Trial in Ovarian cancer cohort (MITO; GSE25204) [73]. The AOCS expression microarray data were retrieved from GEO and processed as described by Choi et al. [17] before analysis. We retained only patients with the serous ovarian cancer subtype that received platinum-based therapy from AOCS (*n* = 226). Outliers were identified using the arrayQualityMetrics R package [97], leaving 224 patients from the AOCS cohort and 107 patients from the MITO cohort for further analysis.

Differential mRNA and miRNA expression analysis results were replicated using the expression auto-cutoff and Kaplan–Meier analysis method used by the KM-Plotter tool [75]. The replication of our network analysis results was performed using the Prognostic Index (PI) estimation method as described by Aguirre-Gamboa et al. in the SurvExpress biomarker validation tool [32]. We used the AOCS and MITO datasets to validate our mRNA and miRNA networks, respectively. Survival plots in Figure 5 were generated using the R package survminer (v. 0.4.8) [98].
